# Supplementary material for: Identification of photoperiod-induced specific miRNAs in the adrenal glands of Sunite sheep (Ovis aries)
Source: Front Vet Sci. 2022 Jul 22;9:888207. doi: 10.3389/fvets.2022.888207 (PMC9354845; doi:10.3389/fvets.2022.888207)
Supplement: Supplementary file 1 [file Data_Sheet_1.ZIP › Supplementary file/miRDeep2 file.zip!/miRDeep2 file/Know_Structure/SP42A2/oar-mir-376e.pdf]

miRBase precursor	: oar-mir-376e
Total read count	: 1024
oar-miR-376e-5p read count	: 259
oar-miR-376e-3p read count	: 506
oar-miR-376b-5p read count	: 259
remaining reads	: 0

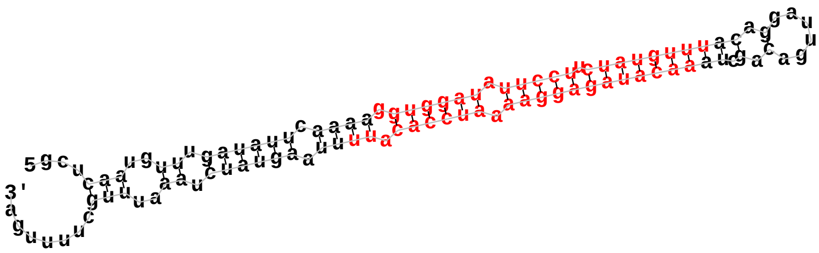

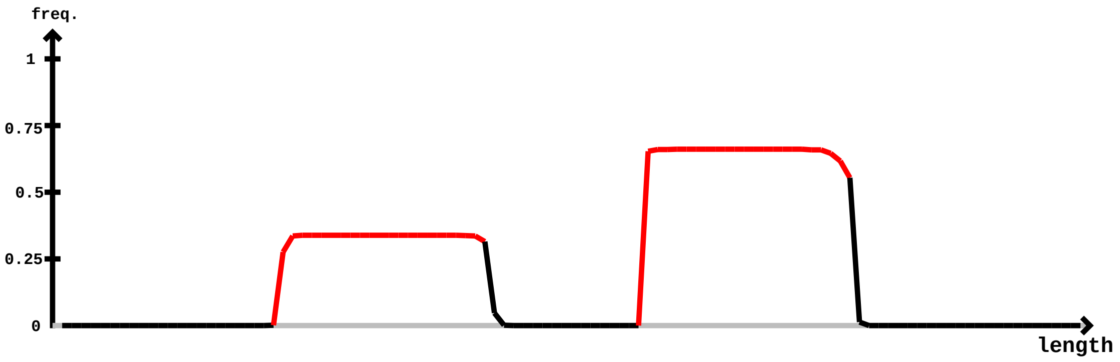


```
                                                            oar-miR-376e-3p
                     oar-miR-376b-5p
                     oar-miR-376e-5p
5'- gcucaauguuugauauucaaaagguggauauuccuucuauguuuacaggauugacagcuaaacauagaggaaaauccacauuuuaaguaucuaaauuugcuuuga   -3'    exp
    ((.....((((((((((.((((.((((((.(((((.((((((((((.(......).).))))))))))))))).)))))).)))).)))))).))))..))......   reads      mm          sample
    .....................agguggauauuccuucuauguuu.............................................................   1          0           seq
    ......................gguggauauuccuucuaugu...............................................................   1          0           seq
    ......................gguggauauuccuucuauguu..............................................................   12         0           seq
    ......................gguggauauuccuucuauguuu.............................................................   172        0           seq
    ......................gguggauauuccuucuauguuua............................................................   25         0           seq
    .......................guggauauuccuucuauguu..............................................................   4          0           seq
    .......................guggauauuccuucuauguuu.............................................................   31         0           seq
    .......................guggauauuccuucuauguuua............................................................   10         0           seq
    .......................guggauauuccuucuauguuuac...........................................................   1          0           seq
    ........................uggauauuccuucuaug................................................................   1          0           seq
    ........................uggauauuccuucuauguuu.............................................................   1          0           seq
    ............................................................aacauGgaggaaaaucc............................   1          1           seq
    ............................................................aacauagaggaaaaucU............................   1          1           seq
    ............................................................aacauagaggaaaauccac..........................   10         0           seq
    ............................................................aacauagaggaaaauccaca.........................   22         0           seq
    ............................................................aacauagaggaaaauccacau........................   47         0           seq
    ............................................................aacauagaggaaaauccacauu.......................   409        0           seq
    ............................................................aacauagaggaaaauccacauuu......................   10         0           seq
    .............................................................acauagaggaaaauccacau........................   1          0           seq
    .............................................................acauagaggaaaauccacauu.......................   4          0           seq
    ...............................................................auagaggaaaauccacauu.......................   1          0           seq
```
